# Case of Prolapsus Ani; Cured Partly by an Excision of a Portion of the Inner Coat of the Intestine, and Partly by the Introduction of a Wax Candle within the Cavity of the Rectum

**Published:** 1800

**Authors:** Thomas Whately

**Affiliations:** Member of the Corporation of Surgeons of London.


XVI. *Case of Prolapsus Ani; cured partly by an Excision of a Portion of the inner Coat of the Intestine, and partly by the Introduction of a Wax Candle within the Cavity of the Rectum. By Mr.* Thomas Whately, *Member of the Corporation of Surgeons of London.*

A Gentleman, about thirty years old, of a very healthy habit of body, and fond of exercise, had been afflicted, for some years, with a troublesome prolapsus ani. The complaint originated, as he conceived, from the violent action of mercurial cathartics. After every evacuation from the rectum, a prolapsus immediately ensued, in size equal to a hen's egg, which he was always under the necessity of reducing with his fingers, to enable him to walk about again. Exercise, even a gentle walk, would bring on some degree of the prolapsus: on these occasions it would be as big as a large cherry; and frequently of the size and shape of two cherries. If his exercise was more severe, it was followed by a more confiderable protrufion. Whenever the prolapfus was occafioned by walking, the friction foon produced a confiderable tendernefs in the part, accompanied with a difcharge of bloody ichor, and fuch extreme pain as prevented his continuing the exercife for any length of time. Under thefe circumftances, he was fcarcely ever able to fit. If he rode on horfeback, the prolapfus took place to a greater degree than when he walked; he was, of courfe, precluded from this kind of exercife.

In this inconvenient and painful fituation, he applied for relief in the fummer of 1798. I fufpected that he could be cured only by an operation; but previous to any attempt of this kind, I propofed a confultation. It was agreed by Mr. Cline, and myfelf, that our patient fhould make trial of a lotion of vitriolated zinc, diffolved in water, accompanied with a bandage and compreffes; and that he fhould take the Peruvian bark. This plan was purfued for feveral months, without any benefit. I received another application from this gentleman, in the fpring of 1799, at which time he was fcarcely able to attend to the common affairs of life.

The

The neceſſity of an operation being now more evident than ever, I wiſhed again to meet Mr. Cline. At this conſultation it was agreed, that the prolapſed part of the inteſtine, as it uſually appeared after exerciſe, might be ſafely cut off with a pair of ſciſſars, by firſt taking hold of it with the polypus forceps. In a few days afterwards, I cut off the portion then prolapſed; it conſiſted of a ſingle protuberance of the ſize of a cherry. No hæmorrhage of any conſequence enſued.

After the operation, our patient was adviſed to keep at home for a week, both to give the parts time to heal, and to aſcertain whether the inflammation, occaſioned by the operation, would not induce a cloſer adheſion of the coats of the inteſtine, and thereby lead to a permanent cure. The patient recovered from the operation without any unfavourable ſymptom. In about ten days he walked abroad as uſual, but the prolapſus appeared again, though not quite ſo bad as before.

Deſirous of being perfectly free from his complaint, and conceiving that a ſecond operation would complete his cure, he ſolicited me to perform it. Accordingly, in about a week after his recovery, I took off another portion of the prolapſed inteſtine, ſomewhat larger than the former one. On the evening of the ſame day, the prolapſus again took place. It was returned within the rectum, but could not be retained in its ſituation, owing to an inflammation with a tenſion and ſwelling of the parts. The following night the patient was very reſtleſs; the prolapſus being continual. On the next day, the protruded inteſtine was returned ſeveral times, but could not be retained a moment, notwithſtanding the patient was in bed the whole day. The ſecond night was paſſed more reſtleſsly than the firſt. He was in conſtant pain, both in the prolapſed and adjoining parts. He now became hot and feveriſh; loſt his appetite; and his pulſe was full, hard, and quicker than natural. On inſpecting the parts, the prolapſed inteſtine was found to be as large as a pullet's egg, of a dark colour, and actually ſphacelated on the ſurface. There was likewiſe an inflamed, tumefied, and tenſe rim, all around the prolapſed inteſtine. This was the ſphincter ani, which acting as a ligature, produced the ſphacelus. I immediately took away twenty ounces of blood from his arm, which was very ſizy. A purgative was adminiſtered, and a ſtrict injunction junction given, to adhere to the antiphlogiſtic regimen. Letting blood almoſt immediately relieved his pain, and leſſened the tenſion of the parts; aware, however, that he could not have permanent relief, unleſs the prolapſed inteſtine could be kept in its natural ſituation, I returned it with an oiled finger, and then applied ſeveral well-made linen compreſſes to the anus, adding a T bandage to retain them in their ſituation; his head and ſhoulders were likewiſe lowered, and his buttocks raiſed; but in defiance of theſe means, the inteſtine came down again within half an hour. The parts were now fomented, and an emollient poultice was applied; and after the operation of the purge, twenty drops of laudanum were given. The next night, (the third after the operation,) he paſſed more quietly, and on the morning after, the tenſion on the ſphincter, as well as on the prolapſed inteſtine, was leſs than it had been on the two preceding days; the latter, however, ſtill continued nearly as much prolapſed as before, and of a chocolate colour.

Fearing the conſequence of its continuing ſo long in this ſtate with the degree of inflammation which ſtill remained, and being ap-

 prehenſive prehenfive that adhefions might take place, and occafion the prolapfus to become permanent, I perfuaded my patient to let me endeavour to keep the rectum in its natural fituation, by means of a tallow candle. For this purpofe, I introduced the lower end of one of a large fize, and about four inches long, (having firft returned the prolapfed part with my fingers,) keeping about half an inch of the candle without the fphincter ani, with a ftring faftened to it, and fecuring it in that fituation by a bandage. As long as the candle remained entire, it kept up the inteftine in part, but it melted in about an hour; after which the prolapfus returned as before. It immediately occurred to me, that if a fubftance lefs foluble, and of the fame fize, fhape, and length as the tallow candle, were paffed *beyond the fphincter ani, and buried within the cavity of the rectum*, it might, by keeping the gut diftended, act as a kind of peffary, and prevent its paffing the fphincter. And if the inteftine could be retained in this fituation for a few days, while it was in an inflamed ftate, it feemed probable that fuch adhefions might take place, as would afterwards effectually prevent a return of the difeafe. Submitting this idea to the patient, I procured his his consent to the introduction of a wax candle for this purpose, intending, if that should dissolve too soon, to introduce a still harder substance, such as sealing wax. In this experiment, as in the former, the candle was not at first suffered to go beyond the sphincter ani, but was retained in its situation by a bandage; but by the peristaltic motion of the bowels, and the pressure of the abdominal muscles, it was expelled, and followed by the prolapsus in a quarter of an hour after its introduction.

As the tightness from the sphincter was considerable, and the prolapsed part was yet large, and remarkably tender to the touch, the intestine was returned within the cavity of the rectum, at each of these operations, with so much difficulty and pain to the patient, that he seemed now desirous of leaving his case to nature, rather than submit to any further attempts to relieve him by art. I persuaded him, however, to submit to another trial, at which I passed the whole of the candle beyond the sphincter.* By thus burying it within the rectum, the prolapsed intestine was retained in its situation, and the

* I had a string fastened to the end of the candle, as in the former trial, by which I could extract it, if necessary.

the patient became easier than he had been since the first operation. An anodyne was administered in the evening, with the design of preventing any evacuation by stool. The patient passed a very good night, and on the following day, the swelling round the verge of the anus had in some degree subsided. Ease, health, and spirits increased every hour. The following evening the anodyne was again repeated, and a strict horizontal position enjoined. On the third day he continued to mend. Nothing now appeared externally but the thread that was fastened to the end of the candle. Neither this nor the intestine, had shewn any disposition to pass the sphincter. On the fifth day from the introduction of the wax candle, (the ninth after the operation,) the patient was so well, that I judged it safe to withdraw the thread; this was accompanied with nothing but the cotton wick of the candle, the wax having been gradually melted by the heat of the parts. A dose of castor oil, to empty the bowels, was then administered. In a few days the patient fully recovered his health and strength, and was soon able to use any degree of exercise, either on horseback or on foot, without any return of the prolapsus, and ftill continues perfectly free from his complaint.

In an obftinate cafe of prolapfus ani, in an adult perfon, like the prefent, it appears to me highly probable, that if every other method fhould fail, a cure might be obtained, by flitting the rectum for an inch and a half, or two inches, from the anus, and with it the fphincter, as in the operation for a fiftula. This might be done by firft puncturing the found fkin and adipofe membrane with a lancet, at the diftance of about half an inch from the anus, and paffing Pott's crooked bif-toury, with one hand, into the puncture, for the purpofe of perforating and afterwards flitting the rectum, while a finger of the other hand might be introduced into its cavity. By this operation fome inflammation would of courfe be excited in the wounded part of the rectum, which would probably extend itfelf, in fome degree, to the adjoining parts. This inflammation, by the time the wound was cicatrized, would produce a clofer adhefion of the lax internal coat of the rectum, (in which part the difeafe is feated,) to its mufcular coat. This would, in all probability, effectually prevent vent its ever falling down in future below the ſphincter, and thus effect a permanent cure.

Soon after this patient was cured, happening to mention the caſe, with the method of treatment, to my friend, Dr. John Sims, he informed me that having a female patient, who had long laboured under a prolapſus ani, which, from the relaxed ſtate of the ſphincter, was conſtantly down; he had conceived the idea of ſupporting the part by means of a globe peſſary introduced within the ſphincter. From ſome circumſtances, the trial was never made, although he had gone ſo far as to get a ſphere of ivory hollowed out, for the ſake of lightneſs, in the ſame manner as the ivory eggs are made. It ſeems probable, that ſuch a contrivance might in ſome caſes prove of conſiderable utility. Such a peſſary would of courſe be expelled with the alvine excretions, and the inconvenience of returning it every time muſt be ſubmitted to; but if it ſhould keep the part from protruding upon exerciſe, a great point would be gained; nor is it altogether improbable, as my friend remarked, that the conſtant friction and preſſure of a hard body, might might, in time, ſo thicken and harden the cellular texture of the gut, as to effect a permanent cure.

